# GRK5 polymorphisms and Postoperative Atrial Fibrillation following Coronary Artery Bypass Graft Surgery

**DOI:** 10.1038/srep12768

**Published:** 2015-08-03

**Authors:** Lu Liu, Lijun Zhang, Minjie Liu, Yanqun Zhang, Xia Han, Zhiqiang Zhang

**Affiliations:** 1 Department of Cardiology, Laiwu People’s Hospital, Laiwu, Shandong Province, China 271100; 2 Department of cardiac surgery, Laiwu People’s Hospital, Laiwu, Shandong Province, China 271100; 3 Department of cadre health care, Laiwu People’s Hospital, Laiwu, Shandong Province, China 271100

## Abstract

Postoperative atrial fibrillation (POAF) is a serious yet common complication after coronary artery bypass grafting (CABG) surgery. Previous study have identified multiple genetic susceptibility loci for POAF susceptibility after CABG, although some studies are under-powered. However, none of these studies have been conducted among Asians. In current study, we aim to systematically evaluated the previous positive findings for POAF susceptibility after CABG among Chinese population, using a large population-based, two-stage, case-cohort study. From a discovery cohort of 1,348 patients, a total of nine independent loci were evaluated. Six significant SNPs were then assessed in a separately collected validation cohort of 2,000 patients. After adjustment for clinical predictors of POAF, two variants in GRK5 gene (rs4752292, and rs11198893) were replicated with significance were replicated in the validation cohort. The ORs for each additional copy of minor allele were 1.32 (95% CI: 1.15–1.50, P = 5.82 × 10^−5^) and 1.47 (95% CI: 1.28–1.69, P = 1.16 × 10^−7^), respectively. In this two-stage independently collected cardiac surgery cohorts, genetic variations in the GRK5 gene are independently associated with POAF risk in patients who undergo CABG surgery in Asians.

Postoperative atrial fibrillation (POAF) is a serious yet common complication, also a known predictor of in-hospital morbidity and short-term survival after coronary artery bypass grafting (CABG) surgery, occurring in 25% to 40% of patients^1^,^2^,^3^,^4^,^5^. Although POAF after on-pump CABG is benign and self-limiting in most cases, in some cases, it can cause stroke or congestive heart failure and may lead to prolonged hospital stay, increased cost, additional concomitant treatment, and worsened prognosis^6^. Some risk factors and possible preventive strategies for POAF have been identified, like echocardiographic parameters, age, number of vessels bypassed, vessel location, net fluid balance on the operative day, POAF score and so on^7^,^8^,^9^,^10^. However, little is known about the molecular basis of POAF susceptibility.

Identification of patients who are at higher risk of POAF using genetic markers might be an important step for prevention of this type of operative complication and may provide important insight into the pathogenesis of AF and new therapeutic strategies for individual patients according to relative risk. Druing last 20 years, researchers have evaluated multiple genetic susceptibility loci for POAF susceptibility after CABG, although some studies are under-powered^11^,^12^,^13^,^14^,^15^,^16^. However, all of these studies have been conducted among people of European ancestry. Considering that race was a surrogate for genetic determinants of POAF susceptibility after CABG due to genetic disparities among racial/ethnic groups^17^,^18^, investigation of previously reported loci in non-European populations may help to evaluate the generalizability of these initial findings and to identify causal variants. Thus, we conducted this study to evaluated the previous positive findings for POAF susceptibility after CABG among Chinese population.

## Materials and Methods

### Subjects

The methods were carried out in “accordance” with the approved guidelines. Patients undergoing primary CABG surgery without planned concurrent valve surgery were enrolled. The exclusion criteria were as follows: prior cardiac surgery; emergency surgical procedure; acute coronary syndrome; prior myocardial infarction; congestive heart failure; significant valvular heart disease; prior implantation of a permanent pacemaker, implantable cardioverter defibrillator, or cardiac resynchronization therapy defibrillator converted to a standard on-pump procedure; and use of class I or class III anti-arrhythmic agents. Also, the patients were excluded if they were not in sinus rhythm during echocardiography. In current study, we first conducted a case-cohort study in 1,348 subsequently patients from the total cohort as the discovering stage. Then, 2,000 individuals of the remaining cohort comprised the replication cohort for our study. Study protocols were approved by respective institutional review boards of Laiwu People’s Hospital, and participants were enrolled after informed written consent was obtained.

### Data collection and end-point definition

An uniform questionnaire was used to collect patient demographics, preoperative and procedural factors, perioperative medication use, postoperative outcomes obtained from patient interview and medical records, and staff interviews. Diagnosis of new onset POAF was based on postoperative electrocardiogram or rhythm strip or documented by at least 2 of the following: progress notes, nursing notes, discharge summary, or change in medication^3^. Trained medical record data abstractors reviewed the medical records of all subjects. To avoid investigators-related biases, all the physicians involved in patients’ care were blinded to the results of the genetic and biochemical analyses. The in-hospital clinical course of all patients, including major and minor postoperative complications were prospectively recorded.

### SNP selection and genotyping

Through a comprehensive retrieval of Pubmed for previous positive findings for POAF susceptibility after CABG, we identified nine independent loci, including four loci in GRK5 gene (rs3740563, rs4752292, rs11198893, and rs10787959), 3 in 4q25 region (rs2200733, rs13143308, rs10033464), 1 in IL6 gene (rs1800795), and 1 in HSP70 gene (rs2227956). All of the nine SNP, which were not in linkage disequilibrium (LD), were evaluated in the discovering stage. DNA was extracted from white blood cells using standard procedures. Genotyping was performed using validated TaqMan assays (Applied Biosystems). PCR product was amplified utilizing 0.9 μM each of the forward primer and reverse primers, 0.2 μM each of the FAM and VIC MGB (minor groove binder) sequence-specific probes, 3 ng DNA, 5.0 mM MgCl_2_, and 1X TaqMan Universal PCR Master Mix containing AmpliTaq Gold DNA Polymerase in a 5.5 μl reaction volume. Both the SNPs had a call rate of greater than 99%. QC concordance for samples was 100%.

### Statistical Analyses

All statistical tests were 2-sided and a *P* value significance threshold of 0.05 was set. All analyses were conducted using SAS, version 9.2 (SAS Institute, Cary, North Carolina). Descriptive statistics of clinical variables are presented as frequency and percentage for categorical variables and mean ± SD or median (interquartile range) for continuous variables.

Hardy–Weinberg equilibrium (HWE) was tested by comparing observed and expected genotype frequencies among controls (x^2^ test). For each of the SNPs, allelic associations with POAF were assessed using logistic regression analyses adjusted for the POAF Risk Index. The same logistic regression model adjusted for the POAF Risk Index was applied in the replication dataset. To assess the overall effect of candidate SNPs, we then conducted a meta-analysis using the weighted Z-score meta-analysis as implemented in METAL (http://www.sph.umich.edu/csg/abecasis/metal).

## Results

Characteristics of the discovery and validation cohorts, stratified by the occurrence of POAF, are shown in Table 1. The mean age of the discovery cohort was 68.0 ± 9.8 years; 287 (21.3%) of the subjects were female. Of the 1,348 patients in this cohort, 405 (30.0%) developed POAF. For the validation cohort, the mean age was 67.2 ± 9.8 years with 22.1% being female, and 630 (31.5%) of the total subject developed POAF. People in the two genotyping stages were generally comparable for BMI, medical history of hypertension, diabetes, COPD, hyperlipidemia, hemodialysis, and preoperative medications of ACEI/ARB, calcium channel blocker, β-blocker, statins, and postoperative medications of HMG-CoA reductase inhibitor and β-blocker discontinued.

A total of nine independent loci which were previously reported to be associated with POAF susceptibility after CABG were included in the current study, including four loci in GRK5 gene (rs3740563, rs4752292, rs11198893, and rs10787959), 3 in 4q25 region (rs2200733, rs13143308, rs10033464), 1 in IL6 gene (rs1800795), and 1 in HSP70 gene (rs2227956). Of these 9 markers, none was found to deviate from HWE (P > 0.05). The estimates of effect of these variants on POAF susceptibility after CABG in discovery stage, adjusted for age and gender are shown in Table 2. The allelic pattern of six SNPs (GRK5 rs3740563, GRK5 rs4752292, GRK5 rs11198893, GRK5 rs10787959, 4q25 rs13143308, and 4q25 rs10033464) was differentially distributed among the the patients who developed POAF and those who didn’t develop POAF, with the p-value being significant (ranged from 6.87 × 10^−6^ to 0.022). The most signigicant association was detected for GRK5 rs11198893 (P = 6.87 × 10^−6^). Each additional copy of minor allele A was associated with a 1.54-fold increased risk of developing POAF (OR = 1.54, 95% CI: 1.27–1.85).

Then, the six significant vanriants were evaluated in a validation cohort with 630 subjects with POAF and 1370 subjects without POAF (Table 3). Two variants in GRK5 gene (rs4752292, and rs11198893) were replicated with significance. For rs4752292, each additional copy of minor allele T was associated with a 1.32-fold increased risk of developing POAF (OR = 1.32, 95% CI: 1.15–1.50, P = 5.82 × 10^−5^). While for rs11198893, each additional copy of minor allele A was associated with a 1.47-fold increased risk of developing POAF (OR = 1.47, 95% CI: 1.28–1.69, P = 1.16 × 10^−7^). Sensitivity analyses were used to evaluated the robustness of these findings by additional adjustments by age, gerder, BMI, medical history, preoperative medications, and postoperative medications were conducted respectively. The results didn’t change materially.

## Discussion

AF is the most frequently occurring arrhythmia both in ambulatory and postcardiac surgical patients and is associated with significant morbidity. There is strong evidence for heritability of POAF. In this large population-based, two-stage, case-cohort study, we idenfied that GRK5 rs4752292, and GRK5 rs11198893 were significantly associated with increased risk of POAF after CABG. Testing for these genetic markers could improve risk stratification and potentially personalize therapy for preventing POAF. To the best of our knowledge, this study is the first to demonstrate that genetic variations in the GRK5 gene are associated with POAF risk in patients who undergo CABG surgery in Asians.

For more than 2 decades, researchers have attempted to identify predictors of AF after cardiac surgery^19^. In the general population, the pathophysiology of AF has been extensively studied, yet it remains incompletely understood^19^,^20^. Similarly, in the context of cardiac surgery, numerous risk factors for POAF have been identified, but the pathophysiology of this entity is still to a large extent elusive^21^,^22^,^23^,^24^. Identification of genetic markers for POAF might be an important step for prevention of this type of operative complication and may provide important insight into the pathogenesis of AF and new therapeutic strategies for individual patients according to relative risk. Previous researchers have identified multiple genetic susceptibility loci for POAF susceptibility after CABG^11^,^12^,^13^,^14^,^15^,^16^, although all of these studies have been conducted among people of European ancestry. In 2003, Gaudino *et al.*^11^ first identified that The −174G/C interleukin-6 polymorphism could influence postoperative interleukin-6 levels and POAF in 110 primary isolated coronary artery bypass patients, which are strong arguments in favor of an inflammatory component of the development of atrial arrhythmias after cardiac surgery. Then many studies evaluated other genetic determinants of POAF^11^,^12^,^13^,^14^,^15^,^16^. These data open new perspectives on a possible genetic determinants of POAF after CABG and suggest a genetic modulation of the postoperative course and outcome after cardiac surgery.

In current study, we first identified genetic variations in the GRK5 gene is associated with POAF risk after CABG in Asians. G protein-coupledreceptor kinase 5 (GRK5), which encodes a member of the guanine nucleotide-binding protein (G protein)-coupled receptor kinase subfamily of the Ser/Thr protein kinase family, was located in chromosome 10q26.11^25^. Phosphorylation of ARRB1 by GRK5 inhibits G-protein independent MAPK1/MAPK3 signaling downstream of 5HT4-receptors and GRK5 potentially acts as a physiological regulator of ű-adrenergic receptor activity^26^,^27^. GRK5 is abundantly expressed in the normal human heart and involves in many biological processes of cardiovascular system^28^,^29^,^30^,^31^,^32^,^33^. Liggett *et al.*^31^ found that a functional loss-of-function SNP (rs17098707) in the coding region of the GRK5 gene has been associated with blunting the effects of ű-adrenergic receptor agonists via enhanced receptor desensitization. Furthermore, Kertai *et al.*^16^ confirmed that genetic variation in GRK5 is associated with POAF despiteperioperative BB therapy in patients undergoing CABG surgery. All of these findings validated the importance of GRK5 gene in the developmet of POAF after CABG.

A strength of this study was the acceptable statistical power to distinguish relatively small genotype associations. Another strength of the current study was a large population, a two-stage genotyping design to minimize type I error. Limitations for this study should be more larger-scale studies are needed to warranted our findings, which may ultimately lead to a comprehensive understanding of the conceivable roles in POAF. Second, the candidate gene design might bias the real situation.

Conclusively, genetic variants in GRK5 gene are identified to be independently associated with an increased risk of POAF. These findings delineate an important genetic role in the etiology of POAF and provide a detailed genomic landscape in which to examine biological mechanisms.

## Additional Information

**How to cite this article**: Liu, L. *et al.* GRK5 polymorphisms and Postoperative Atrial Fibrillation following Coronary Artery Bypass Graft Surgery. *Sci. Rep.* **5**, 12768; doi: 10.1038/srep12768 (2015).

## Figures and Tables

**Table 1:** Demographic Characteristics of the Discovery and Validation Cohorts.

Demographic	Discovery Cohort			Validation Cohor		
	POAF (N = 405)	No (N = 943)	POAF P	POAF (N = 630)	No (N = 1,370)	POAF P
Age, y	68.5 ± 9.6	67.8 ± 9.9	0.230	67.4 ± 10.3	67.1 ± 9.6	0.526
Gender (% female)	87 (21.6%)	200 (21.2%)	0.911	143 (22.7%)	300 (21.9%)	0.689
BMI, kg/m ^2^	24.3 ± 4.3	24.8 ± 4.7	0.067	24.3 ± 4.3	24.5 ± 4.7	0.364
Medical history						
Hypertension	324 (80.0%)	725 (76.9%)	0.207	530 (84.1%)	1,140 (83.2.%)	0.608
Diabetes	104 (25.7%)	225 (23.9%)	0.476	158 (25.1%)	326 (23.8%)	0.534
COPD	22 (5.5%)	44 (4.7%)	0.550	37 (5.8%)	78 (5.7%)	0.873
Hyperlipidemia	201 (49.7%)	433 (45.9%)	0.211	290 (46.1%)	629 (45.9%)	0.960
Hemodialysis	53 (13.0%)	110 (11.7%)	0.463	80 (12.7%)	156 (11.4%)	0.398
Preoperative medications						
ACEI/ARB	196 (48.4%)	440 (46.7%)	0.558	299 (47.4%)	638 (46.6%)	0.711
Calcium channel blocker	66 (16.3%)	140 (14.8%)	0.498	102 (16.2%)	216 (15.8%)	0.810
β-blocker	319 (78.8%)	736 (78.1%)	0.770	515 (81.7%)	1,133 (82.7%)	0.603
Statins	96 (23.7%)	234 (24.8%)	0.664	143 (22.7%)	326 (23.8%)	0.591
Postoperative medications						
HMG-CoA reductase inhibitor	304 (75.1%)	730 (77.4%)	0.349	484 (76.8%)	1,059 (77.3%)	0.815
β-blocker discontinued	11 (2.7%)	33 (3.5%)	0.458	22 (3.5%)	53 (3.9%)	0.681

**Table 2:** Logistic Regression Analysis of Genetic Predictors of Postoperative Atrial Fibrillation in the Study Populations (Stage I).

				Discovery Cohort			
SNP	Gene/loci	Location	Alleles*	MAF (AF)	MAF (No AF)	OR (95% CI)	P
rs3740563	GRK5	Chr10:121095400	C/A	0.27	0.22	1.31 (1.09–1.58)	**0.005**
rs4752292	GRK5	Chr10:121100153	G/T	0.35	0.29	1.32 (1.11–1.57)	**1.98 × 10**^−**3**^
rs11198893	GRK5	Chr10:121107900	G/A	0.29	0.21	1.54 (1.27–1.85)	**6.87 × 10**^−**6**^
rs10787959	GRK5	Chr10:121131313	G/A	0.47	0.42	1.22 (1.04–1.44)	**0.016**
rs2200733	4q25	Chr4:111929618	C/T	0.42	0.39	1.13 (0.96–1.34)	0.145
rs13143308	4q25	Chr4:111933868	T/G	0.27	0.32	0.79 (0.65–0.94)	**9.73 × 10**^−**3**^
rs10033464	4q25	Chr4:111940210	G/T	0.25	0.21	1.25 (1.03–1.52)	**0.022**
rs1800795	IL6	Chr7:22727026	G/C	0.07	0.06	1.18 (0.85–1.64)	0.327
rs2227956	HSP70	Chr6:31810495	T/C	0.21	0.19	1.13 (0.92–1.39)	0.230

^*^Major allele/minor allele.

P value in bold means statistically significant.

**Table 3:** Logistic Regression Analysis of Genetic Predictors of Postoperative Atrial Fibrillation in the Study Populations (Stage I and II).

SNP	Gene/Loci	Alleles	Stages	OR (95% CI)	P
rs3740563	GRK5	C/A	Discovery	1.31 (1.09–1.58)	0.005
			Validation	1.21 (0.89–1.38)	0.089
rs4752292	GRK5	G/T	Discovery	1.32 (1.11–1.57)	**1.98 × 10**^−**3**^
			Validation	1.31 (1.07–1.63)	**0.012**
			Total	1.32 (1.15-1.50)	**5.82 × 10**^−**5**^
rs11198893	GRK5	G/A	Discovery	1.54 (1.27–1.85)	**6.87 × 10**^−**6**^
			Validation	1.38 (1.01–1.56)	**0.004**
			Total	1.47 (1.28–1.69)	**1.16 × 10**^−**7**^
rs10787959	GRK5	G/A	Discovery	1.22 (1.04–1.44)	0.016
			Validation	0.97 (0.84-1.21)	0.744
rs13143308	4q25	T/G	Discovery	0.79 (0.65–0.94)	9.73 × 10^−3^
			Validation	1.17 (0.99–1.32)	0.032
rs10033464	4q25	G/T	Discovery	1.25 (1.03–1.52)	0.022
			Validation	1.05 (0.87–1.33)	0.652

## References

[b1] Bramer S. *et al.* Body mass index predicts new-onset atrial fibrillation after cardiac surgery. Eur J Cardiothorac Surg. 40, 1185–1190 (2011). 21450475 10.1016/j.ejcts.2011.02.043

[b2] Silva R. G., Lima G. G., Guerra N., Bigolin A. V. & Petersen L. C. Risk index proposal to predict atrial fibrillation after cardiac surgery. Revista brasileira de cirurgia cardiovascular : orgao oficial da Sociedade Brasileira de Cirurgia Cardiovascular. 25, 183–189 (2010). 20802909 10.1590/s0102-76382010000200009

[b3] Mathew J. P. *et al.* A multicenter risk index for atrial fibrillation after cardiac surgery. Jama. 291, 1720–1729 (2004). 15082699 10.1001/jama.291.14.1720

[b4] Banach M. *et al.* Postoperative atrial fibrillation - what do we really know? Curr Vasc Pharmacol. 8, 553–572 (2010). 19538179 10.2174/157016110791330807

[b5] Mariscalco G. *et al.* Atrial fibrillation after isolated coronary surgery affects late survival. Circulation. 118, 1612–1618 (2008). 18824644 10.1161/CIRCULATIONAHA.108.777789

[b6] Fujiwara M. *et al.* Prediction of atrial fibrillation after off-pump coronary artery bypass grafting using preoperative total atrial conduction time determined on tissue Doppler imaging. Circ J. 78, 345–352 (2014). 24284884 10.1253/circj.cj-13-0900

[b7] Krohn B. G. Useful understanding of postoperative atrial fibrillation. Circulation. 100, 1250 (1999). 10515706

[b8] Caretta Q. *et al.* Ventricular conduction defects and atrial fibrillation after coronary artery bypass grafting. Multivariate analysis of preoperative, intraoperative and postoperative variables. Eur Heart J. 12, 1107–1111 (1991). 1782937 10.1093/oxfordjournals.eurheartj.a059845

[b9] Selzer A. & Walter R. M. Adequacy of preoperative digitalis therapy in controlling ventricular rate in postoperative atrial fibrillation. *Circulation*. 34, 119–122 (1966). 5940234 10.1161/01.cir.34.1.119

[b10] Mariscalco G. *et al.* Bedside tool for predicting the risk of postoperative atrial fibrillation after cardiac surgery: the POAF score. Journal of the American Heart Association. 3, e000752 (2014). 24663335 10.1161/JAHA.113.000752 PMC4187480

[b11] Gaudino M. *et al.* The −174G/C interleukin-6 polymorphism influences postoperative interleukin-6 levels and postoperative atrial fibrillation. Is atrial fibrillation an inflammatory complication? Circulation. 108 **Suppl 1**, II195–199 (2003). 12970232 10.1161/01.cir.0000087441.48566.0d

[b12] Afzal A. R. *et al.* Association of Met439Thr substitution in heat shock protein 70 gene with postoperative atrial fibrillation and serum HSP70 protein levels. *Cardiology*. 110, 45–52 (2008). 17934269 10.1159/000109406

[b13] Plante I., Fournier D., Mathieu P. & Daleau P. A pilot study to estimate the feasibility of assessing the relationships between polymorphisms in hKv1.5 and atrial fibrillation in patients following coronary artery bypass graft surgery. Can J Cardiol. 24, 41–44 (2008). 18209767 10.1016/s0828-282x(08)70546-4 PMC2631247

[b14] Body S. C. *et al.* Variation in the 4q25 chromosomal locus predicts atrial fibrillation after coronary artery bypass graft surgery. Circ Cardiovasc Genet. 2, 499–506 (2009). 20031626 10.1161/CIRCGENETICS.109.849075 PMC2801871

[b15] Virani S. S. *et al.* Usefulness of single nucleotide polymorphism in chromosome 4q25 to predict in-hospital and long-term development of atrial fibrillation and survival in patients undergoing coronary artery bypass grafting. Am J Cardiol. 107, 1504–1509 (2011). 21414601 10.1016/j.amjcard.2011.01.026 PMC3087849

[b16] Kertai M. D. *et al.* G Protein-Coupled Receptor Kinase 5 Gene Polymorphisms Are Associated with Postoperative Atrial Fibrillation Following Coronary Artery Bypass Graft Surgery in Patients Receiving Beta-Blockers. Circ Cardiovasc Genet. 7, 625–33 (2014). 25049040 10.1161/CIRCGENETICS.113.000451 PMC4270923

[b17] Rader F. *et al.* Influence of race on atrial fibrillation after cardiac surgery. Circulation. Arrhythmia and electrophysiology. 4, 644–652 (2011). 21841189 10.1161/CIRCEP.111.962670 PMC3201706

[b18] Nazeri A. *et al.* Race/ethnicity and the incidence of new-onset atrial fibrillation after isolated coronary artery bypass surgery. *Heart Rhythm*. 7, 1458–1463 (2010). 20620230 10.1016/j.hrthm.2010.06.037

[b19] Chua S. K., Shyu K. G. & Lo H. M. Postoperative atrial fibrillation and cardiac surgery. Circ J. 78, 265 (2014). 24257102 10.1253/circj.cj-13-1352

[b20] Cleland J. G., Joseph A. & Pellicori P. Fish oil vs olive oil for postoperative atrial fibrillation. Jama. 309, 871 (2013). 10.1001/jama.2013.670 23462774

[b21] Imazio M. *et al.* Colchicine for prevention of postpericardiotomy syndrome and postoperative atrial fibrillation: the COPPS-2 randomized clinical trial. Jama. 312, 1016–1023 (2014). 25172965 10.1001/jama.2014.11026

[b22] Chua S. K. *et al.* Association Between Renal Function, Diastolic Dysfunction, and Postoperative Atrial Fibrillation Following Cardiac Surgery. Circ J. 77, 2303–10 (2013). 23979566

[b23] Akaishi M. Intravenous infusion of ultra-short-acting beta-blocker for postoperative atrial fibrillation is the one of choice. Circ J. 76, 1083–1084 (2012). 22361919 10.1253/circj.cj-12-0142

[b24] Mearns B. M. Atrial fibrillation: Colchicine lowers postoperative AF risk. Nat Rev Cardiol. 9, 67 (2012). 10.1038/nrcardio.2011.196 22143082

[b25] Kunapuli P., Onorato J. J., Hosey M. M. & Benovic J. L. Expression, purification, and characterization of the G protein-coupled receptor kinase GRK5. J Biol Chem. 269, 1099–1105 (1994). 8288567

[b26] Kunapuli P., Gurevich V. V. & Benovic J. L. Phospholipid-stimulated autophosphorylation activates the G protein-coupled receptor kinase GRK5. J Biol Chem. 269, 10209–10212 (1994). 8144599

[b27] Premont R. T., Koch W. J., Inglese J. & Lefkowitz R. J. Identification, purification, and characterization of GRK5, a member of the family of G protein-coupled receptor kinases. *J Biol Chem*. 269, 6832–6841 (1994). 8120045

[b28] Philipp M., Berger I. M., Just S. & Caron M. G. Overlapping and Opposing Functions of G Protein-coupled Receptor Kinase 2 (GRK2) and GRK5 during Heart Development. J Biol Chem. 289, 26119–26130 (2014). 25104355 10.1074/jbc.M114.551952 PMC4176219

[b29] Haack K. K. *et al.* Parallel changes in neuronal AT1R and GRK5 expression following exercise training in heart failure. Hypertension. 60, 354–361 (2012). 22753221 10.1161/HYPERTENSIONAHA.112.195693 PMC3409876

[b30] Eijgelsheim M., Visser L. E., Uitterlinden A. G. & Stricker B. H. Protective effect of a GRK5 polymorphism on heart failure and its interaction with beta-adrenergic receptor antagonists. Pharmacogenomics. 9, 1551–1555 (2008). 18855542 10.2217/14622416.9.10.1551

[b31] Liggett S. B. *et al.* A GRK5 polymorphism that inhibits beta-adrenergic receptor signaling is protective in heart failure. Nat Med. 14, 510–517 (2008). 18425130 10.1038/nm1750 PMC2596476

[b32] Oyama N. *et al.* Chronic beta-adrenergic receptor stimulation enhances the expression of G-Protein coupled receptor kinases, GRK2 and GRK5, in both the heart and peripheral lymphocytes. Circ J. 69, 987–990 (2005). 16041172 10.1253/circj.69.987

[b33] Yi X. P., Gerdes A. M. & Li F. Myocyte redistribution of GRK2 and GRK5 in hypertensive, heart-failure-prone rats. Hypertension. 39, 1058–1063 (2002). 12052842 10.1161/01.hyp.0000019130.09167.3b

